# Ancient nuclear genome duplication events in vertebrates facilitated the functional diversification of ohnologous cytochrome *c*: insights from *Buergeria* frogs

**DOI:** 10.1093/molbev/msag243

**Published:** 2026-09-25

**Authors:** Yuka Asaeda, Sotaro Fujii, Hajime Ogino, Michael A Hough, Takeshi Igawa, Yoshihiro Sambongi

**Affiliations:** Graduate School of Integrated Sciences for Life, Hiroshima University, Higashi-Hiroshima 739-8528, Japan; Amphibian Research Center, Hiroshima University, Higashi-Hiroshima 739-8526, Japan; Graduate School of Integrated Sciences for Life, Hiroshima University, Higashi-Hiroshima 739-8528, Japan; Graduate School of Integrated Sciences for Life, Hiroshima University, Higashi-Hiroshima 739-8528, Japan; Amphibian Research Center, Hiroshima University, Higashi-Hiroshima 739-8526, Japan; Diamond Light Source, Harwell Science and Innovation Campus, Didcot OX11 0DE, UK; Graduate School of Integrated Sciences for Life, Hiroshima University, Higashi-Hiroshima 739-8528, Japan; Amphibian Research Center, Hiroshima University, Higashi-Hiroshima 739-8526, Japan; Seto Inland Sea Carbon-Neutral Research Center, Hiroshima University, Higashi-Hiroshima 739-8528, Japan; Faculty of Human Environmental Studies, Hiroshima Shudo University, Hiroshima 731-3195, Japan

**Keywords:** *Buergeria* frog, H_2_O_2_ resistance, genome duplication, heme, testis-type cytochrome *c*

## Abstract

In vertebrates, a nuclear gene encoding mitochondrial cytochrome *c* (CYC) was duplicated and diverged into one with ubiquitous expression (somatic-type) and the other with testis-preferred expression (testis-type). In this study, synteny-based phylogenetic analyses suggest that *cyc* ohnologs originated through whole-genome duplication events in the common ancestor of vertebrates and have been conserved for approximately 450 to 540 million years. Molecular evolutionary analyses revealed pervasive purifying selection and an approximate Phe/Tyr dichotomy at position 46 between somatic- and testis-type CYC proteins. This was the case in 2 closely related amphibians, *Buergeria buergeri* and its hot-spring-dwelling species, *Buergeria japonica*. The recombinant testis-type CYC proteins from *B. buergeri* (Bb-CYCT) and *B. japonica* (Bj-CYCT) exhibited significantly higher resistance to hydrogen peroxide (H_2_O_2_) than somatic-type counterpart proteins (Bb-CYCS and Bj-CYCS). Structural analysis showed that the Tyr-46 residues in the testis-type CYC proteins face the heme, suggesting a protective role against H_2_O_2_ through oxidation of its hydroxyl group compared to the spatially corresponding Phe-46 residue in the somatic-type counterparts. When the Tyr-46 residue in Bj-CYCT was replaced with the Phe residue, the H_2_O_2_ resistance was significantly reduced, confirming the functional importance of Tyr-46. These results demonstrate that functional divergence between somatic- and testis-type *Buergeria* CYC proteins is at least partly attributable to the Phe-to-Tyr substitution, suggesting that ancient nuclear genome duplication contributed to tissue-specific divergence of mitochondrial function.

## Graphical Abstract

**Graphical Abstract:**
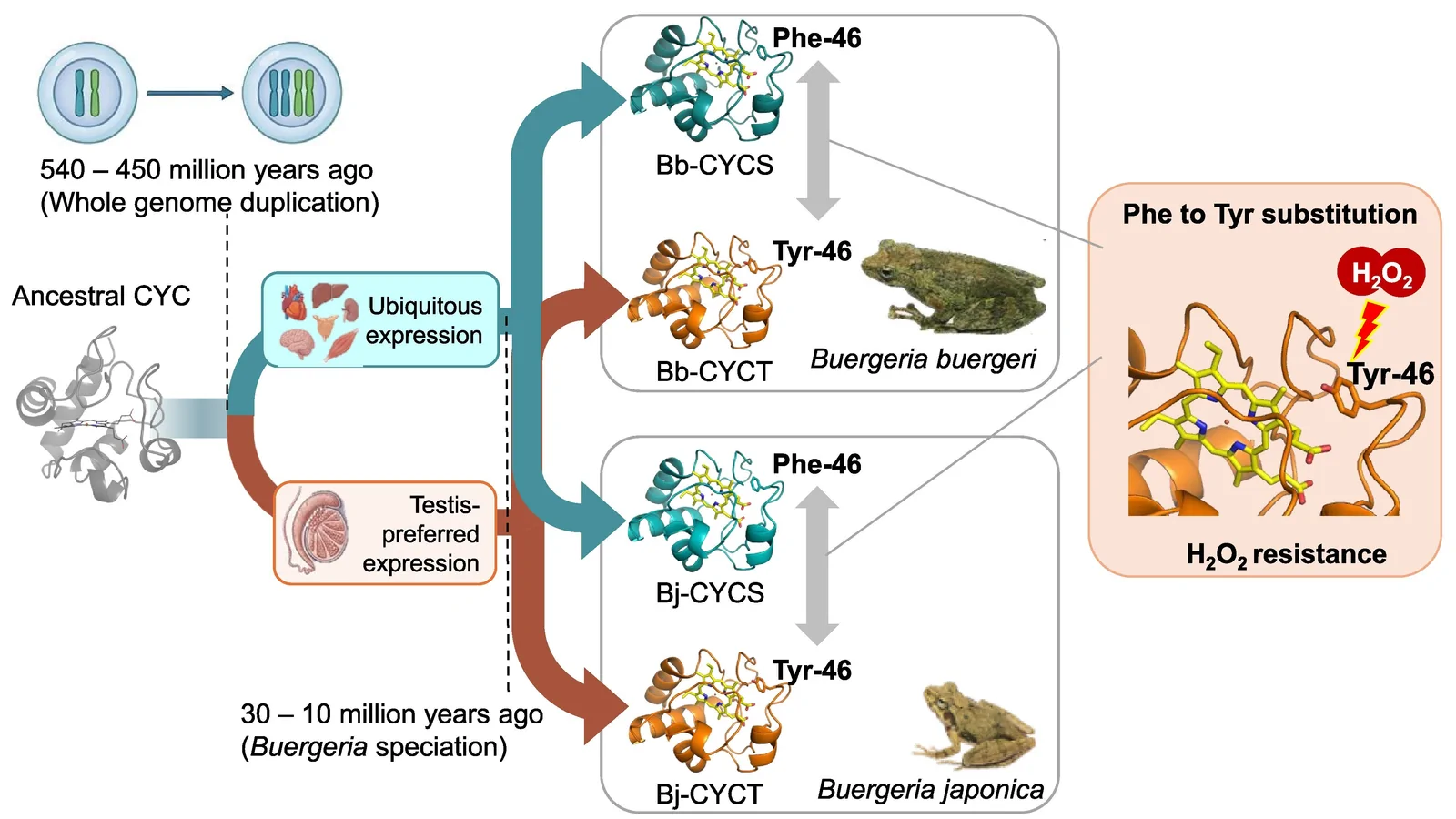

Highlights

- Vertebrate *cyc* genes diverged following ancient nuclear genome duplication
- A Phe-to-Tyr substitution confers H_2_O_2_ resistance in the *Buergeria* CYC proteins
- The H_2_O_2_-resistant *cyc* is preferentially expressed in the testis
- The Tyr-46 residue responsible for this resistance faces the heme

## Introduction

During vertebrate evolution, the phenomenon of whole genome duplication (WGD) has facilitated diversification of species by enabling the neofunctionalization, specialization, and subfunctionalization of the resulting duplicated genes, known as ohnologs (Ohno 1970; Cañestro et al. 2013). However, such evolutionary processes have been investigated with a focus on developmental genes, and much less is known about genes involved in the primary metabolic process, including those associated with organelles. The mitochondrion, an organelle originating from an ancient endosymbiosis of an α-proteobacterial ancestor, is the primary site of ATP production in eukaryotic cells (Kurland and Andersson 2000; Dyall et al. 2004). During the course of organelle evolution, most genes of the endosymbiont were transferred to the host nucleus, resulting in a mitonuclear system in which respiratory chain complexes are encoded by both mitochondrial and nuclear genomes (Wallace 2007; Vafai and Mootha 2012).

To our best knowledge, nuclear-encoded mitochondrial cytochrome *c* (CYC) oxidase subunits in vertebrates diversified through the WGD, and their tissue-specific expression patterns have been investigated (Little et al. 2010). Among the nuclear-encoded components of the electron transport chain, CYC plays a pivotal role as the sole electron carrier from the cytochrome *bc*_1_ complex to cytochrome *c* oxidase, sustaining oxidative phosphorylation (Crofts 2004; Kadenbach and Hüttemann 2015; Yoshikawa and Shimada 2015). In addition, CYC exhibits peroxidase-like activity that contributes to the mitigation of oxidative damage (Hannibal et al. 2016). Some vertebrates possess 2 types of CYC: one ubiquitously expressed in somatic tissues (somatic-type) and the other preferentially expressed in testis (testis-type) (Hennig 1975; Goldberg et al. 1977). In frogs such as *Xenopus* species, genome database searches have revealed the existence of both somatic- and testis-type *cyc* genes (*cycs* and *cyct*, respectively) (Hellsten et al. 2010). Although the presence of 2 types of CYC hints at their biological significance, the evolutionary and functional aspects of the CYC divergence remain poorly understood.

Comparative genome analysis has revealed that ancestral-type nuclear chromosomal synteny is more conserved in *Xenopus tropicalis* and *Gallus gallus* than in *Homo sapiens* (Hellsten et al. 2010). Therefore, amphibians are well-suited for understanding the functional divergence of metabolic genes following the vertebrate WGD events. We have analyzed biological aspects of 2 closely related frogs: *Buergeria buergeri*, which lives in ambient mountain streams (Nishizawa et al. 2011), and *Buergeria japonica*, surviving in hot-spring environments (Tominaga et al. 2015; Komaki et al. 2016; Asaeda et al. 2023; Priambodo et al. 2024). As *B. buergeri* and *B. japonica* share a common ancestor, they may possess suitable CYC proteins for comparative analyses, as previously demonstrated for bacterial CYC proteins (Sambongi et al. 2002).

In this study, by means of synteny-based phylogenetic analyses, we first illustrated that *cyc* genes were duplicated through the ancient WGD events during vertebrate evolution. Molecular evolutionary and structural analyses showed dichotomic amino acid differences between somatic- and testis-type CYC proteins at a residue facing the heme. We then characterized the biochemical properties of testis-type CYC proteins from *B. buergeri* (Bb-CYCT) and *B. japonica* (Bj-CYCT), together with their somatically expressed counterparts (Bb-CYCS and Bj-CYCS). The testis-type CYC proteins showed resistance to oxidative stress. The results obtained provide insights into the functional divergence of duplicated *cyc* genes in vertebrates.

## Results

### Synteny-based phylogenetic analyses

Comparative synteny analysis of *cyc* genes in chordates revealed that the syntenic regions containing *cycs* and *cyct* include *Hox* clusters (*cycs* near the *hoxa* cluster and *cyct* near the *hoxd* cluster), in which orthologous *cycs* and *cyct* were widely conserved (Fig. 1a). Except for rodents and primates, which are thought to have lost either one or both copies of *cyc*, at least 1 synteny region including *Hox* clusters was traced back to elephant shark and lamprey, a basal lineage of vertebrates. Although the *Ciona* genome contains 2 *cyc* genes that appear to have arisen through tandem duplication, no synteny was observed.

**Figure 1:**
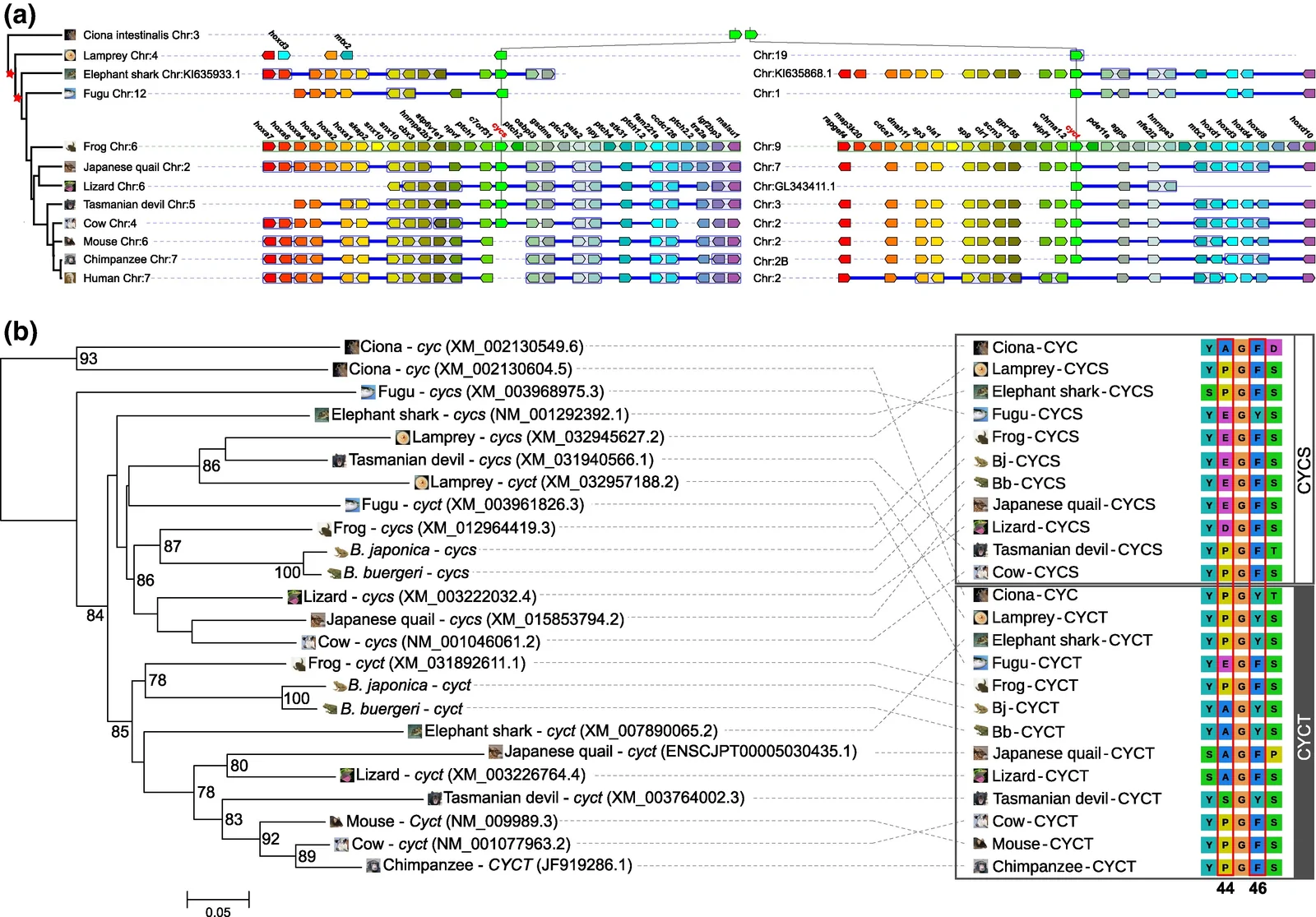
Synteny-based phylogenetic analyses across vertebrates. a) Syntenic conservation of *cycs* and *cyct* genes across vertebrates. Genomic synteny information and graphical output were obtained from Genomicus v110.01 (Nguyen et al. 2022) and adapted for this figure. Adapted with permission from the Institut de Biologie de l’ENS (IBENS), École normale supérieure–PSL. Each horizontal track represents a genomic region from the indicated species. Genes are shown as arrow-shaped boxes, with arrow direction indicating transcriptional orientation. The arrangement of corresponding genes across species illustrates conserved gene order within syntenic regions. Solid horizontal lines represent chromosomal or scaffold segments. Background boxes indicate groups of neighboring genes assigned by Genomicus to conserved syntenic blocks. Vertical connecting lines indicate orthologous relationships of *cyc* genes among species. Gene names are shown above the *X. tropicalis* (“Frog”) synteny map, including *cycs* and *cyct*. Chromosome names are indicated on the left of each track. Species are arranged according to the vertebrate phylogenetic tree (Gee 2015). Asterisks on the phylogenetic tree indicate the two WGD events. b) A neighbor-joining phylogenetic tree of orthologous *cyc* gene sequences identified from the synteny map. Numbers at nodes indicate bootstrap support values (%) based on 500 replicates; only values ≥75% are shown. Branch lengths are proportional to the number of nucleotide substitutions per site. Species names are followed by gene names (*cyc*, *cycs*, and *cyct*) and GenBank/Ensembl accession numbers in parentheses. The right panel shows a multiple sequence alignment of the region surrounding positions 44 and 46, which are boxed. CYC protein sequences in the alignment panel are arranged according to the species order shown in (a).

A BLAST search of the *Buergeria* larval transcriptome data using the *X. tropicalis cycs* and *cyct* sequences identified *B. buergeri* and *B. japonica* orthologs, *Bb-CYCS*, *Bb-CYCT*, *Bj-CYCS*, and *Bj-CYCT*, respectively. Molecular phylogenetic analysis using the full-length coding nucleotide sequences, including those appearing in Fig. 1a exhibited that somatic- and testis-type *cyc* genes (*cycs* and *cyct*, respectively) formed distinct clades, with the exception of *Ciona*, lamprey, and fugu (Fig. 1b). This dichotomic pattern is also confirmed between somatic- and testis-type *cyc* genes of *B. buergeri* and *B. japonica*. Although the codon corresponding to amino acid position 44 was identified by the mixed effects model of evolution (MEME) as being subject to episodic diversifying selection (*P* = 0.021), the neighboring codon corresponding to position 46 showed an approximate Phe/Tyr dichotomy between CYCS and CYCT, including the *Buergeria* proteins (Fig. 1b, right). Fixed effect site-level (FEL) analysis detected significant evidence of pervasive purifying selection at this codon (β < α, *P* = 0.0129), suggesting that further amino acid-altering substitutions have been selectively suppressed after the divergence of the 2 ohnologs. Contrast-FEL analysis further indicated no significant difference in selective pressure at this site between the somatic- and testis-type *cyc* lineages, with estimated dN/dS ratios below 1 in both lineage groups. These results suggest that the 46th codon has been functionally constrained within each *cyc* type.

### Amino acid sequence comparison among *Buergeria* CYC proteins

We next examined the 4 *Buergeria* CYC proteins at the amino acid level (Fig. 2a). Within each of the *Buergeria* species, the ohnolog pairs show substantial divergence: Bb-CYCT and Bb-CYCS differ by 16 amino acid residues (84% sequence identity), and Bj-CYCT and Bj-CYCS differ by 13 amino acid residues (85% sequence identity) (Fig. 2a). In contrast, comparisons between orthologous CYC proteins reveal much higher conservation: the 2 testis-type CYC proteins from both species, Bb-CYCT and Bj-CYCT, differ by only 4 amino acid residues (96% sequence identity), and the somatic-type CYC proteins, Bb-CYCS and Bj-CYCS, differ by only 1 residue (99% sequence identity) (Fig. 2a). These results indicate that the ohnolog pairs are more diverged than the ortholog pairs in *Buergeria* species. In addition, from the ortholog comparisons (Fig. 2a), the testis-type orthologs show slightly greater amino acid divergence than the somatic-type orthologs in the 2 *Buergeria* species, although the basis for this difference remains unclear.

**Figure 2:**
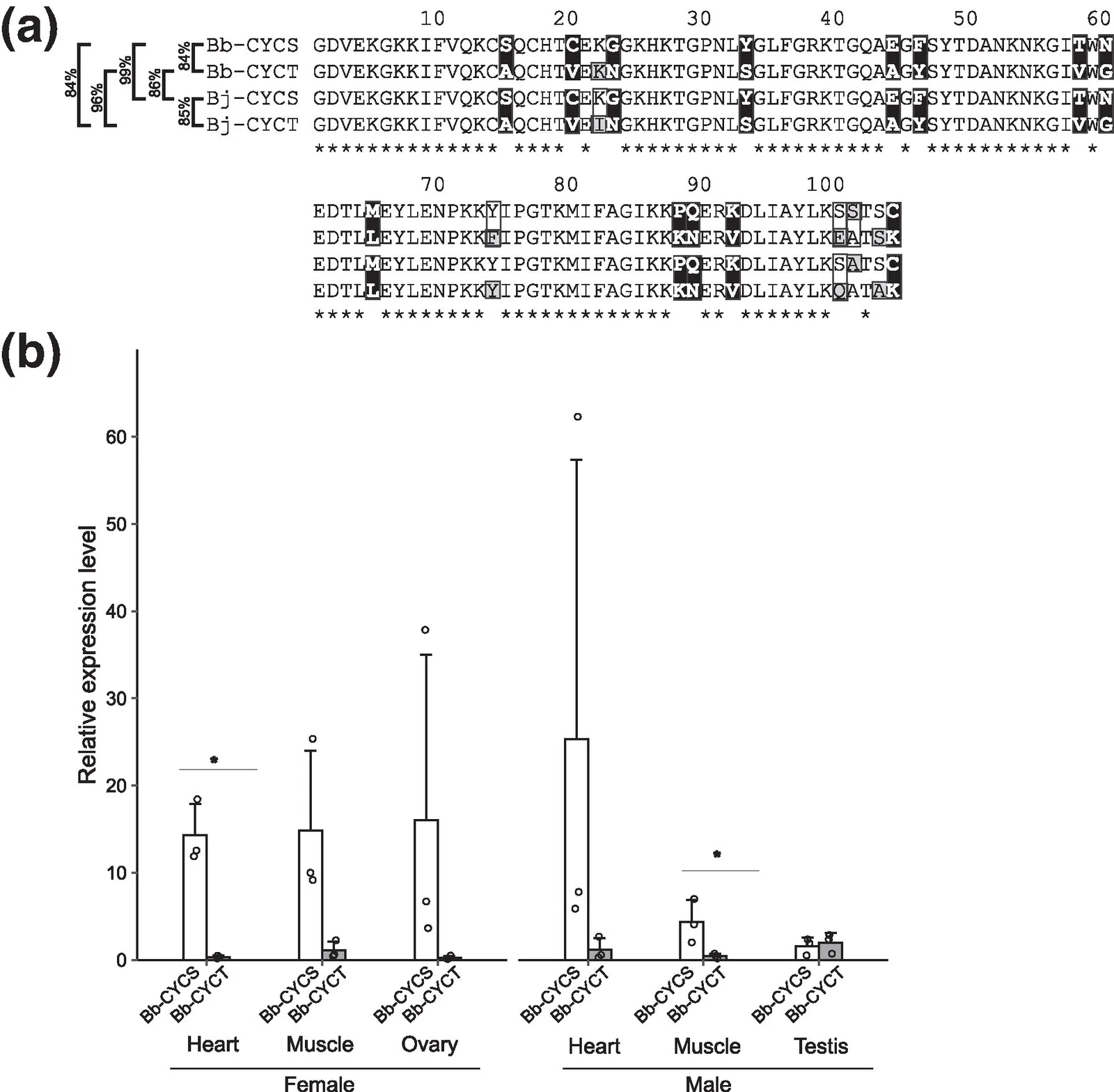
Amino acid sequence alignment of somatic- and testis-type CYC proteins from *Buergeria* species and their gene expression levels. a) Comparison between somatic- and testis-type *cyc* ohnologous and *B. buergeri* and *B. japonica cyc* orthologous gene products in each of the *Buergeria* species. The commonly different amino acid residues are shadowed. The different amino acid residues within only 1 species are boxed. The different amino acid residues between orthologs are gray-boxed. The sequence identities are also shown. b) Expression levels of *Bb-CYCS* and *Bb-CYCT* genes relative to *tuba4a* gene in heart, muscle, ovary, and testis of *B. buergeri*. The qRT-PCR data from *B. buergeri* males and females were collected (*N* = 3). The asterisk indicates a statistically significant difference by *t*-test (*: *P* < 0.05). The error bars indicate the standard deviation. The expression level of the *tuba4a* gene was used as a reference for each biological replicate. The dots represent individual data.

### Expression levels of *cycs* and *cyct* in *B. buergeri*

The relative expression levels of *cycs* and *cyct* of *B. buergeri* (*Bb-CYCS* and *Bb-CYCT*, respectively) in heart, muscle, ovary, and testis were measured by quantitative real-time (qRT)-PCR. In the male testis, expression of *Bb-CYCT* was observed, and its level was comparable to or higher than that of *Bb-CYCS* (Fig. 2b). The expression of *Bb-CYCT* was also observed in other tissues of both female and male, but their levels were apparently lower than those in the male testis. In contrast, *Bb-CYCS* expression was observed across somatic tissues such as heart, muscle, and ovary. Consistently, the transcriptome analysis of *X. tropicalis* male and female adults showed that *cyct* was predominantly expressed in testis, with weak expression in other somatic tissues, while *cycs* was broadly expressed in multiple tissues (Figure S1). These results indicate that *cyct* genes, including *Bb-CYCT* were preferentially expressed in testis.

### AlphaFold modeling of Bj-CYCS, Bb-CYCS, Bb-CYCT, and Bj-CYCT

To investigate the structural differences potentially associated with functional differences among the *Buergeria* CYC proteins, we then applied AlphaFold3 structure modeling. The AlphaFold3 models of the Bj-CYCS, Bj-CYCT, Bb-CYCS, and Bb-CYCT exhibited a globular structure with 5 α-helices surrounding a covalently bound heme (Fig. 3), with high reliability based on predicted local distance difference test values for most amino acid residues.

**Figure 3:**
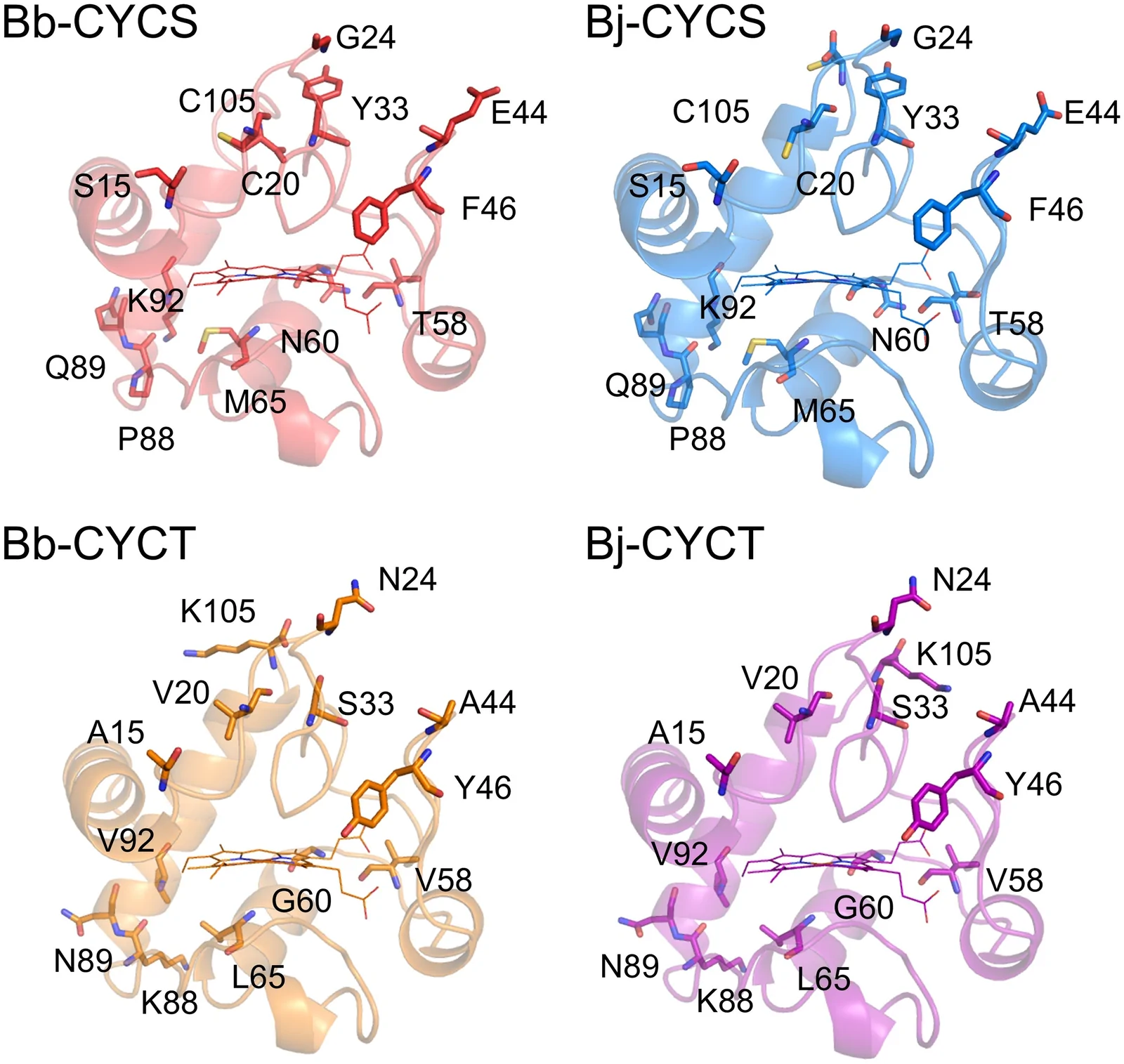
Structural comparison among Bb-CYCT, Bb-CYCS, Bj-CYCT, and Bj-CYCS simulated by AlphaFold3. The main chain and heme structures are shown as ribbon and stick models, respectively. The commonly different 13 amino acid residues among 4 CYC proteins are shown as stick models.

Of the 13 amino acid residues that differed between *Buergeria* somatic- and testis-type CYC proteins, all except for the 46th residue were located on the protein surface in the structural models (Fig. 3). The 46th residue was located near the heme; at this position, hydrophobic Phe found in somatic-type CYC proteins was replaced by more polar Tyr in testis-type CYC proteins (Fig. 3).

### Recombinant protein expression

For the biochemical characterization, 4 recombinant *Buergeria* CYC proteins (Bb-CYCS, Bb-CYCT, Bj-CYCS, and Bj-CYCT) were expressed in the periplasm of *Escherichia coli* JCB387 cells co-transformed with the pEC86 plasmid. The expression of CYC holoprotein was visually confirmed by the pinkish coloration of the osmotic fluids. Sodium dodecyl sulfate-polyacrylamide gel electrophoresis (SDS-PAGE) analysis after purification steps showed single major bands around 10 kDa, corresponding to the expected molecular mass of each CYC protein (Figure S2). The N-terminal sequences of all 4 purified CYC proteins were determined as Gly-Asp-Val-Glu-Lys- (also see Fig. 2a), verifying the correct cleavage of the artificially fused signal peptide and the proper maturation of CYC proteins in the *E. coli* cells. These results indicate that not only bacterial CYC proteins but also *Buergeria* mitochondrial CYC can be exogenously produced in the same *E. coli* expression system in addition to plant chloroplast CYC (Kojima et al. 2005). The *Buergeria* CYC proteins successfully produced were used for structural and biochemical analyses described below.

### Crystal structure of Bj-CYCS and its heme environment

A crystal structure of Bj-CYCS was determined at a resolution of 2.48 Å. The protein exhibited a globular structure with 5 α-helices surrounding a covalently bound heme (Fig. 4a). Structural comparison between Bj-CYCS and other CYC proteins based on Cα atoms produced root-mean-square deviation (RMSD) values less than 1 Å (Table S1), indicating that the overall CYC structures among various organisms are highly conserved. The heme is covalently attached to the polypeptide of Bj-CYCS via thioether bonds with Cys-14 and Cys-17, as verified from the electron density map (Fig. 4a). The Fe atom of the heme was coordinated by the side chains of His-18 and Met-80 with bond lengths to coordinating atoms of 2.1 Å and 2.4 Å, respectively, forming a typical hexacoordinate heme (Fig. 4a).

**Figure 4:**
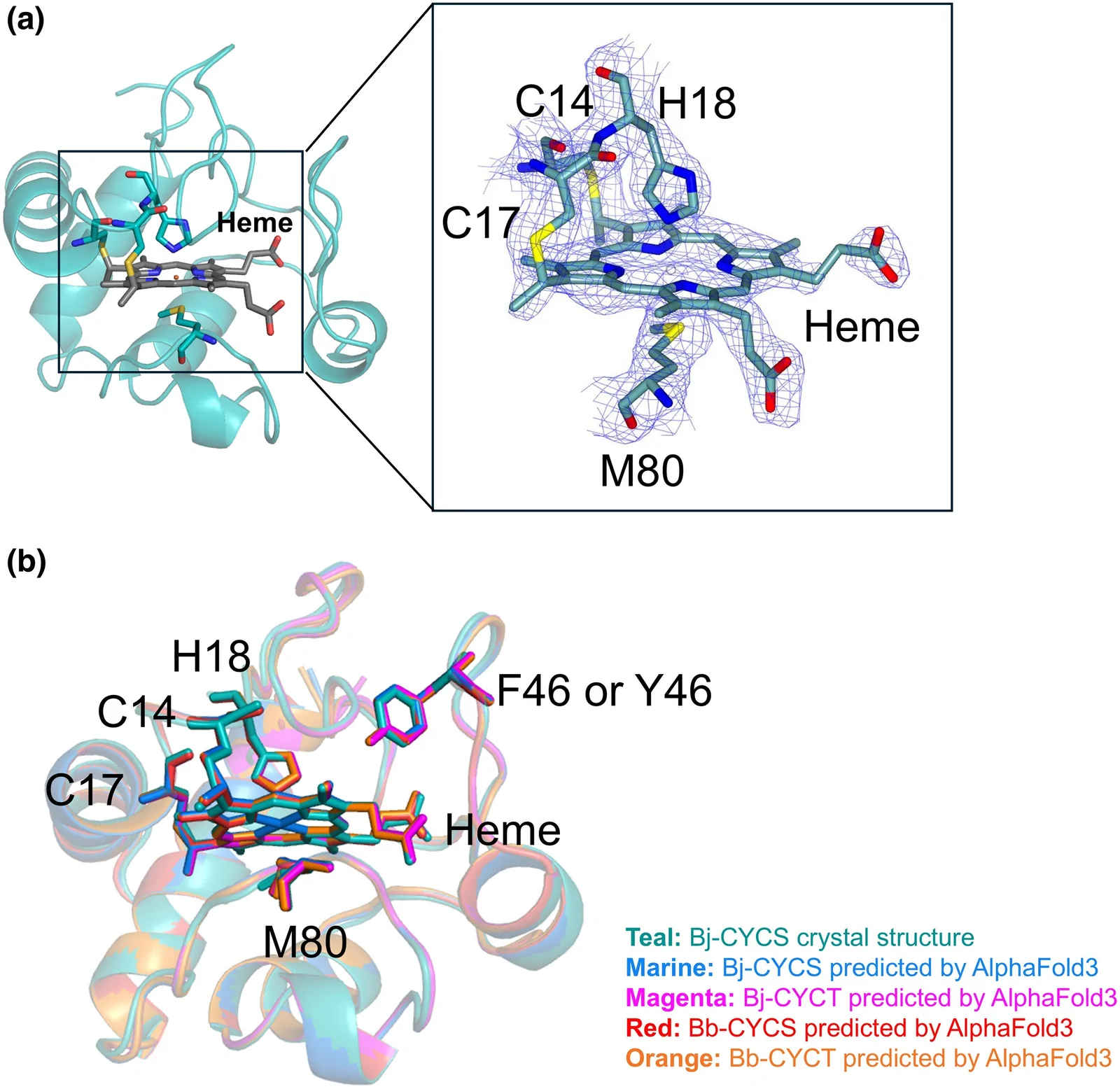
Crystal structure of Bj-CYCS and comparison with AlphaFold3 models. a) Overall X-ray crystal structure (PDB ID: 24OQ). The main chain and heme structures are shown as ribbon and stick models, respectively. 2*F*_o_-*F*_c_ electron density map around the heme is also shown in the close-up view. b) Superposition of the AlphaFold3-predicted models and the crystal structure of Bj-CYCS.

The crystal structure of Bj-CYCS and all AlphaFold3 models superimposed well (Fig. 4b), with RMSD values based on Cα atoms being less than 1 Å. In addition, the heme-binding and -surrounding residues, Cys-14, Cys-17, His-18, Phe/Tyr-46, and Met-80, were well aligned among the AlphaFold3-predicted and crystal structures (Fig. 4b), suggesting that the AlphaFold3 models reliably captured the heme architecture.

### Spectroscopic properties, thermal denaturation, and redox potential analyses

All 4 recombinant *Buergeria* CYC proteins exhibited UV-visible absorption spectra similar to those of typical CYC. In the air-oxidized state, a Soret peak was observed at approximately 409 nm, which was shifted to 412 nm upon reduction of all CYC proteins (Fig. 5a). The reduction simultaneously exhibited an increase in absorption at 550 nm, although minor peaks at the same wavelength remained in some oxidized states, presumably because of residual reduced state. These results indicate the presence of a hexacoordinated heme structure as observed in the simulated structures by AlphaFold3 described above. The circular dichroism (CD) spectra showed 2 signals at 208 and 222 nm for all CYC proteins (Fig. 5b), which are the characteristic features of α-helical proteins and consistent with previous studies of CYC (Sambongi et al. 2002).

**Figure 5:**
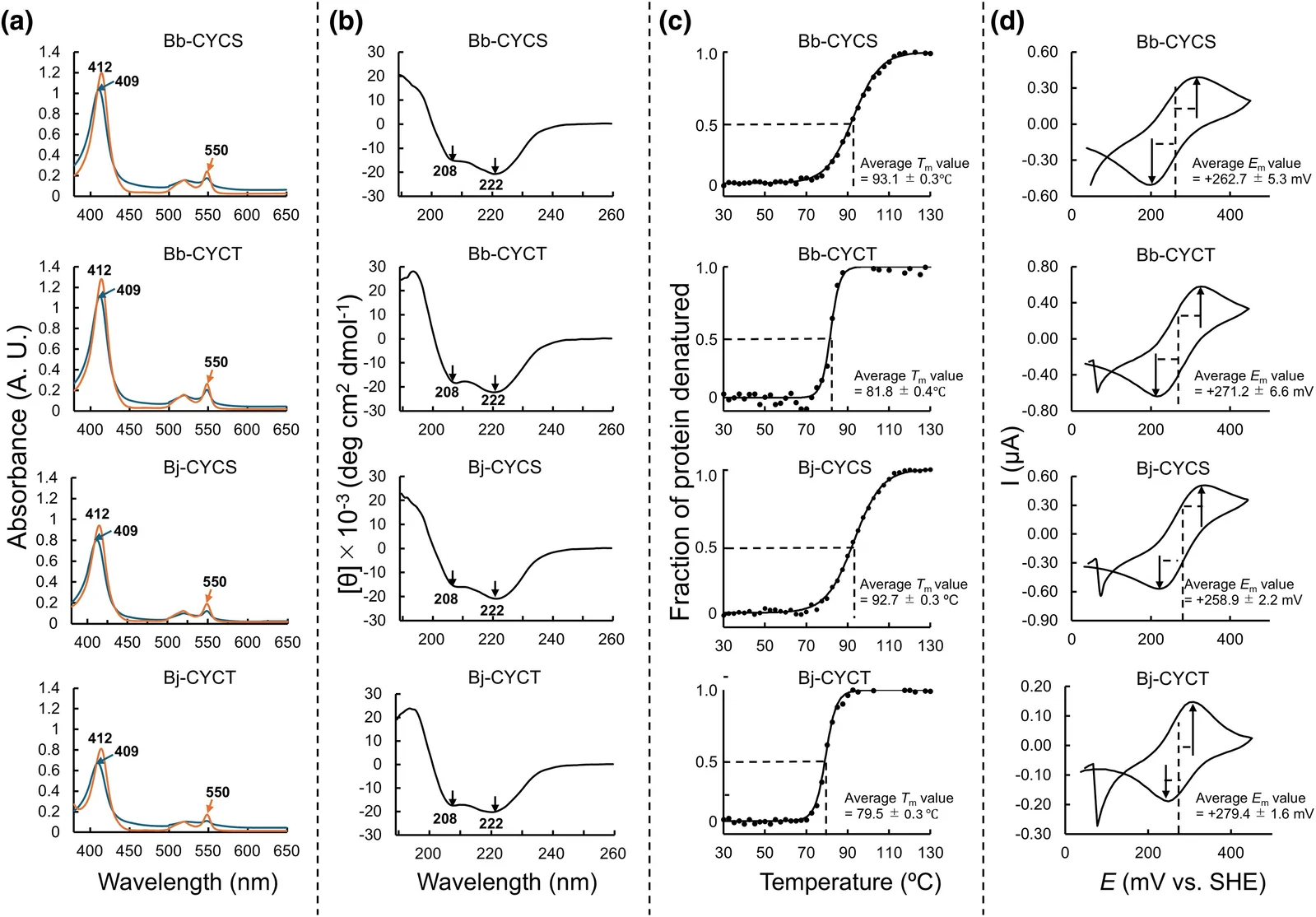
Spectroscopic and electrochemical properties of the recombinant somatic- and testis-type CYC proteins from *Buergeria* species. a) Visible absorption spectra of the 4 proteins at 25 °C. Spectra of oxidized and reduced CYC proteins are shown. The oxidized proteins exhibit a Soret peak at approximately 409 nm, whereas the reduced proteins exhibit a Soret peak at approximately 412 nm together with increased absorption at 550 nm. Relevant wavelengths are indicated by arrows. b) CD spectra of the 4 proteins at 25 °C. Relevant wavelengths are indicated by arrows. c) Normalized thermal denaturation curves by CD analysis based on CD ellipticity at 222 nm. The fractions of representative raw data are shown in circles at a temperature interval of 2.5 °C. The fitting curves are also shown in solid black lines. Each test was performed 5 times. d) Cyclic voltammogram by CV analysis. The representative cyclic voltammogram for each protein is shown. Each test was performed 3 times.

Thermal denaturation of the 4 CYC proteins was measured by CD ellipticity at 222 nm. The normalized denaturation curves were fitted as a two-state model (Fig. 5c), suggesting that all the recombinant CYC proteins undergo a transition between the native and denatured states and cooperative unfolding without a detectable intermediate. The *T*_m_ values, defined as midpoints of the denaturation transition, were as follows: Bb-CYCS, 93.1 ± 0.3 °C; Bj-CYCS, 92.7 ± 0.3 °C; Bb-CYCT, 81.8 ± 0.4 °C; and Bj-CYCT, 79.5 ± 0.3 °C (means and standard deviations) from 5 independent measurements.

The electrochemical properties of the proteins were characterized by cyclic voltammetry for the 4 CYC proteins (Fig. 5d). The *E*_m_ values, calculated from the average of oxidation and reduction peaks, were as follows: Bb-CYCS, +262.7 ± 5.3 mV; Bj-CYCS, +258.9 ± 2.2 mV; Bb-CYCT, +271.2 ± 6.6 mV; and Bj-CYCT, +279.4 ± 1.6 mV (means and standard deviations) from 3 independent measurements.

### Hydrogen peroxide resistance

The hydrogen peroxide (H_2_O_2_) resistance of the 4 recombinant *Buergeria* CYC proteins was evaluated by measuring the decrease in the Soret band absorbance at 408 nm in the presence of 3 mM H_2_O_2_. All the 4 CYC proteins exhibited a time-dependent loss of absorbance, as shown in the case of Bj-CYCS (Fig. 6a), indicating heme degradation as described previously (Liu et al. 2006). From the normalized curves of the absorbance at 408 nm for all CYC proteins (Fig. 6b), the half-life times of heme degradation were calculated as follows: Bj-CYCS, 8.5 ± 0.0 min; Bb-CYCS, 9.3 ± 0.3 min; Bb-CYCT, 18.8 ± 3.4 min; and Bj-CYCT, 30.5 ± 5.7 min (means and standard deviations) from 3 independent measurements, showing that Bj-CYCT and Bb-CYCT have significantly slower heme degradation rate than Bj-CYCS and Bb-CYCS. These results indicate that testis-type CYC proteins, Bb-CYCT and Bj-CYCT, have higher resistance to H_2_O_2_ than the somatic counterparts, Bb-CYCS and Bj-CYCS.

**Figure 6:**
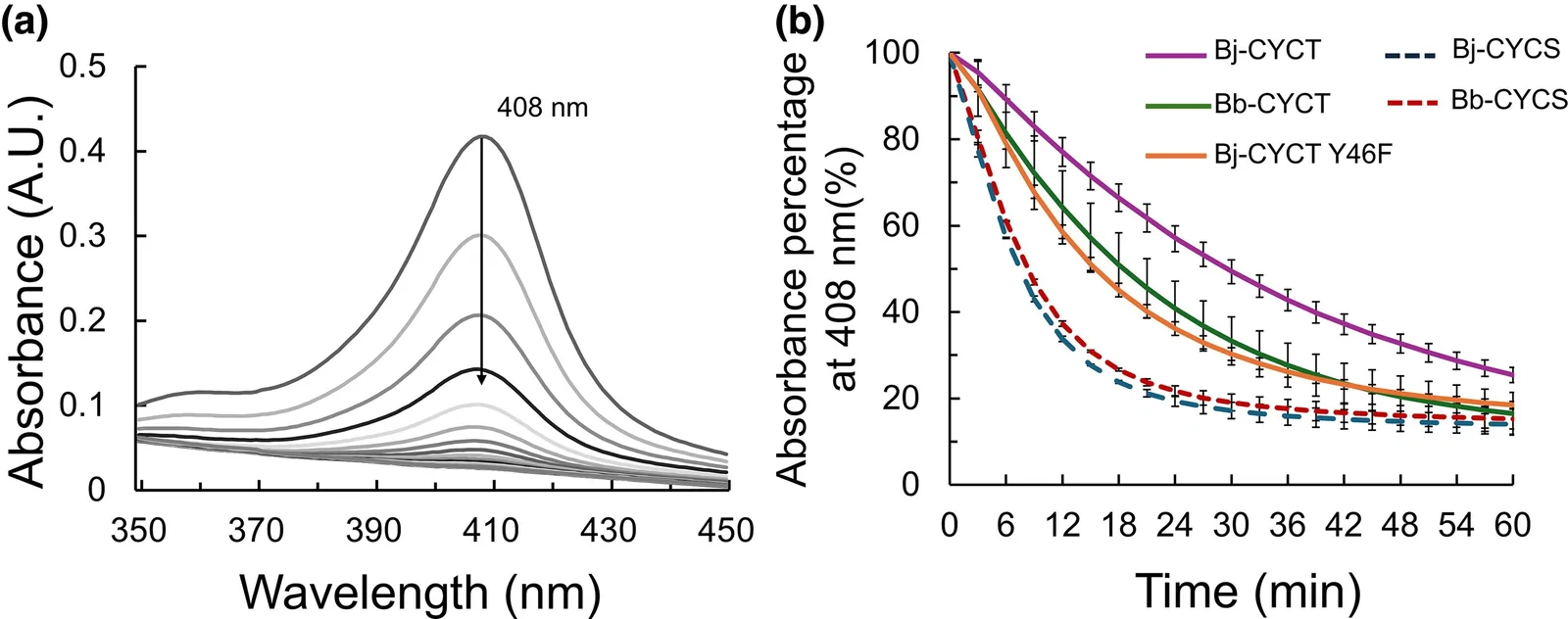
H_2_O_2_-induced heme degradation. a) Representative absorbance of the oxidized Bj-CYCS in the presence of 3 mM H_2_O_2_. The arrow indicates the time-course shift with spectral scanning every 3 min. b) Comparison of degradation percentage among Bb-CYCS, Bb-CYCT, Bj-CYCS, Bj-CYCT, and Bj-CYCT Y46F variant in the presence of 3 mM H_2_O_2_. Each test was performed 3 times. The error bars indicate the standard deviation.

### Hydrogen peroxide resistance of Bj-CYCT Y46F variant

To verify the structural insights into the role of the Tyr-46 residue as discussed above, the H_2_O_2_ resistance of the Bj-CYCT Y46F variant was assessed. The Y46F mutation introduced had only a minor effect on the absorption and CD spectra, thermal denaturation profile, and redox potential value (Figure S3). The Soret band of absorbance at 408 nm in the Bj-CYCT Y46F variant also exhibited a time-dependent decrease in absorbance in the presence of 3 mM H_2_O_2_, similar to the Bj-CYCS wild-type protein (Fig. 6a). However, the half-life time of the heme degradation of Bj-CYCT Y46F variant was 16.0 ± 0.6 min, which was significantly shorter than that of the Bj-CYCT wild-type (30.4 min) although heme degradation proceeded more slowly than in wild-type Bj-CYCS (8.5 min) (Fig. 6b). This demonstrates that the Tyr-46 residue around the heme of Bj-CYCT is partially responsible for the H_2_O_2_ resistance, which possibly appears to be the case in Bb-CYCT because they exhibit high structural similarity as discussed above (Fig. 3).

## Discussion

The present synteny-based phylogenetic analyses reveal that orthologs of *cycs* and *cyct* are found near *hoxa* and *hoxd* clusters, respectively (Fig. 1a). The hox clusters are known as typical ohnologs which have arisen through the 2 rounds of WGD (Lee et al. 2006; Lynch and Wagner 2009; Ravi et al. 2009). Thus, the 2 *cyc* genes that were located in the syntenic regions are consistent with their origin as ohnologs during ancient vertebrate WGD events. As the second WGD occurred in the common ancestor leading to vertebrates, the origin of *cyc* ohnologs can be traced back to 540 to 450 million years ago (Holland and Ocampo Daza 2018). Such a long-term preservation of the *cyc* ohnologs in most vertebrate groups underscores their functional significance. This may relate to the increased oxygen concentration during the Cambrian period (Hu et al. 2025), when the second WGD occurred, which in turn strengthens the importance of WGD for the evolutionary processes in oxidative phosphorylation, not only for those associated with development (see Introduction).

Consistent with the conserved synteny observed among amphibians (Sun et al. 2015; Bredeson et al. 2024; Chen et al. 2024), preliminary inspection of our unpublished draft genome assemblies also suggested that both *cyct* and *cycs* are present as distinct genomic loci in the 2 *Buergeria* species. Because these draft genome assemblies are not yet publicly available, we treat this observation only as supporting evidence for the genomic presence of both *cyc* copies in *Buergeria*. Their interpretation as ohnologs is based primarily on the conserved synteny and phylogenetic evidence presented above. The speciation into *B. japonica* and *B. buergeri* from their common ancestor occurred from 30 to 10 million years ago (Nishizawa et al. 2011; Tominaga et al. 2015), marking the beginning of orthologous *cyc* divergence. Therefore, it is plausible that the duration of orthologous *cyc* divergence in the 2 *Buergeria* species is less than that of ohnologous divergence, resulting in the amino acid sequence differences between the *Buergeria* ohnologs and orthologs (Fig. 2a).

In this study, Bb-CYCS, Bb-CYCT, Bj-CYCS, and Bj-CYCT were biochemically analyzed; they have typical features in terms of visible absorption and CD spectra, two-state thermal denaturation profile, and redox potential (Fig. 5) that have already been observed in other CYC proteins (Takeda et al. 2009; Masanari et al. 2014). A new biochemical finding is the role of the Tyr-46 residue in the H_2_O_2_ resistance of testis-type CYC proteins from the 2 *Buergeria* species compared to the somatic counterparts having Phe residues at the corresponding position (Figs 3 and 6). The hydroxyl group (-OH) of the Tyr residue may act as a sacrificial electron sink by forming an -•OH radical that intercepts oxidizing equivalents derived from H_2_O_2_ (Tomášková et al. 2021), thereby possibly buffering oxidative attack on the porphyrin ring. Thus, the difference between Tyr and Phe in the *Buergeria* CYC proteins partially could affect the H_2_O_2_ resistance. Consistently, in horse heart CYC and myoglobin, the Tyr residue around the heme, although spatially different from that of the *Buergeria* residue, can form radical species, also resulting in the high H_2_O_2_ resistance (Liao et al. 2016; Tomášková et al. 2021). The enhanced H_2_O_2_ resistance of Bj-CYCT may be advantageous in the potentially oxidative environment of the testis and sperm cells. *Buergeria* testis-type CYC may have undergone evolutionary adaptation to cope with testicular reactive oxygen species, including H_2_O_2_, in which the testis is under higher oxidative stress conditions than the somatic tissues (Asadi et al. 2017).

The present biochemical interpretation regarding the H_2_O_2_ resistance of *Buergeria* testis-type CYC is based only on in vitro assays, which may not fully capture physiological roles of testis-type CYC that would function under the complex testis conditions in vivo. However, the present in vitro assays also reveal additional biochemical differences between testis- and somatic-type CYC proteins in addition to their difference in H_2_O_2_ resistance as discussed above. For instance, testis-type CYC proteins have lower *T*_m_ values but higher denaturation cooperativity than those of somatic ones, and the former have higher redox potential values than those of the latter (Fig. 5). Bj-CYCT exhibits the greatest H_2_O_2_ resistance among the 4 CYC proteins (Fig. 6). Such additional biochemical differences will offer a chance to understand the physiological roles of testis-type CYC in the future. As the 2 *Buergeria* species used in this study live in thermally different niches, environmental adaptation of CYC derived from each species will also be of interest, as is discussed in bacterial CYC (Sambongi et al. 2002).

## Materials and methods

### Animals used in this study

Larvae and adults of *B. buergeri* were collected from Takayama River, Hiroshima prefecture. *Buergeria japonica* male and female adults were collected from Seranma Hot Spring, Kuchinoshima Island, Kagoshima Prefecture, and their larvae were bred in the laboratory. All procedures were approved by the Hiroshima University Animal Research Committee (Approval number: G17-9).

### Transcriptome analysis

For the *cyc* gene sequencing analysis, total RNA was extracted from 3 larvae of each *B. buergeri* and *B. japonica* at stage 30 based on *the Rana japonica* developmental stage table (Tahara 1974) using the RNeasy Mini Kit (QIAGEN, Germany) after temporal preservation in RNAlater (QIAGEN, Germany). After genomic DNA was eliminated using the TURBO DNA-free Kit (Ambion, USA), RNA quality and concentration were assessed using a NanoDrop 2000 spectrophotometer (ThermoFisher Scientific, USA), and samples were stored at −80 °C until further analysis.

The extracted RNA samples were subjected to RNA sequencing through a contract service provided by Veritas Corporation. Library preparation was performed using the TruSeq Stranded mRNA Library Preparation Kit (Illumina, USA). Sequencing was conducted on the NovaSeq 6000 platform (Illumina, USA) with 150 bp paired-end reads, generating approximately 6 GB of sequence data per sample.

Quality control and adapter trimming were carried out using FastQC 0.12.1 (https://www.bioinformatics.babraham.ac.uk/projects/fastqc/) and BBDuk, which is included in the BBMap 36.32 package (https://sourceforge.net/projects/bbmap/). After trimming, de novo transcriptome assembly was performed with Trinity 2.15.1 (https://github.com/trinityrnaseq/) (Grabherr et al. 2011; Haas et al. 2013), followed by prediction of coding sequences and amino acid sequences using TransDecoder 5.5.0 (https://github.com/TransDecoder). The assembled transcripts were annotated by comparison to *X. tropicalis* protein sequences (GCF_000004195.4) (Hellsten et al. 2010; Mitros et al. 2019), and orthologs of the genes encoding somatic and testis-type *cyc* were identified using BLAST + 2.16.0 (BLASTP, ≥85% identity; https://blast.ncbi.nlm.nih.gov/doc/blast-help/downloadblastdata.html#downloadblastdata) (Camacho et al. 2009). Transcript abundance was quantified, and gene-level expression estimates were aggregated using Salmon (Patro et al. 2017). The transcriptome sequencing data from *B. buergeri* and *B. japonica* larvae have been deposited in the DNA data bank of Japan (DDBJ) sequence read archive under accession numbers DRR911195 and DRR911196.

### Synteny-based phylogenetic and codon site-model analyses

To trace the evolutionary trajectory of the *cyc* gene, we first identified orthologs within the syntenic regions of *cycs* and *cyct* from the Western clawed frog (*X. tropicalis*) across various chordate species using the Genomicus (version 110.01) (Nguyen et al. 2022), a synteny browser based on comparative genomics. The species examined included sea squirt (*Ciona intestinalis*), sea lamprey (*Petromyzon marinus*), elephant shark (*Callorhinchus milii*), fugu (*Takifugu rubripes*), western clawed frog (*X. tropicalis*), green anole (*Anolis carolinensis*), Japanese quail (*Coturnix japonica*), cattle (*Bos taurus*), mouse (*Mus musculus*), chimpanzee (*Pan troglodytes*), and human (*Homo sapiens*). For *Ciona* and the sea lamprey, syntenic regions were reconstructed by referring to the latest genomic datasets from NCBI (GCF_018327825.1 and GCF_048934315.1, respectively). Synteny maps were generated using the Genomicus database (https://www.genomicus.bio.ens.psl.eu/genomicus-100.01/cgi-bin/search.pl).

Coding sequences of the *cyc* orthologs identified from the synteny map were collected for codon-based multiple sequence alignment and molecular phylogenetic analysis (https://doi.org/10.6084/m9.figshare.32801979). Gene sequences of somatic- and testis-type *Buergeria* CYC proteins were obtained from the present transcriptomic analysis of *Buergeria* larvae as described above. The codon alignment was generated based on the corresponding amino acid alignment and manually inspected to maintain the reading frame. Phylogenetic relationships among *cyc* sequences were inferred using a codon-based distance approach implemented in HyPhy/Datamonkey (Kosakovsky Pond et al. 2020; Kim et al. 2026). Pairwise codon distances were estimated under the Muse–Gaut 1994 codon substitution model crossed with a general reversible nucleotide substitution model (MG94 + REV), and a neighbor-joining tree (Saitou and Nei 1987) was constructed from the resulting codon-based distance matrix (Muse and Gaut 1994). To evaluate nodal robustness, branch support was assessed independently using IQ-TREE3 under codon substitution models with 500 bootstrap replicates (Wong et al. 2026). Bootstrap support values obtained from IQ-TREE3 were mapped onto the HyPhy/Datamonkey MG94 + REV neighbor-joining topology and are shown at the corresponding internal nodes.

To examine whether episodic diversifying selection acted on specific codon sites during *cyc* evolution, we further performed a MEME analysis (Murrell et al. 2012), as implemented in HyPhy/Datamonkey, using the same codon alignment and phylogenetic topology. MEME allows the nonsynonymous/synonymous substitution rate ratio to vary among both sites and branches, thereby detecting codon sites that have experienced episodic positive selection on a subset of lineages. To search for differences in selective pressure at individual and branch sites, FEL model and Contrast-FEL analyses were also conducted (Kosakovsky Pond and Frost 2005; Kosakovsky Pond et al. 2021). Sites with *P* < 0.05 were considered candidates for episodic diversifying or purifying selection.

### Quantitative real-time PCR

Total RNA was extracted from the heart, muscle, and testis of 3 *B. buergeri* adult males and the heart, muscle, and ovary of 3 *B. buergeri* adult females using the RNeasy Mini Kit (QIAGEN, Germany). The cDNA was synthesized from the purified RNA using ReverTra Ace qPCR RT Master Mix with gDNA Remover (TOYOBO, Japan) according to the manufacturer's instructions.

Primers for the amplification of *Bb-CYCS*, *Bb-CYCT*, and *tuba4a* (*alpha*-*tubulin*) genes (Table S2) were designed using Geneious (version 9.1.8). The specificity of the primers for *Bb-CYCS*, *Bb-CYCT*, and *tuba4a* genes, which ranged from 150 to 220 bp was checked by agarose gel electrophoresis. The quality of RNA samples used is shown in Table S3.

The qRT-PCR was conducted using the KOD SYBR qPCR Mix (TOYOBO, Japan) kit, and the resulting cDNA samples were based on the manufacturer's protocol using the Crono STAR 96 real-time PCR system (TaKaRa, Japan). The thermal conditions for the qRT-PCR were set as follows: 2 min at 98 °C for pre-denaturation, 40 cycles of 10 s at 98 °C, 10 s at 60 °C, and 30 s at 68 °C. Relative expression levels of the target genes were calculated using a comparative 2^−ΔCq^ method, with the expression level of the *tuba4a* gene taken as the endogenous reference for each biological replicate. Means with standard deviations of the 2^−ΔCq^ values were obtained from 3 biological replicates. The *C*_t_ values from qRT-PCR analysis were shown in Table S4.

### Protein structure prediction

The three-dimensional structures of Bb-CYCS, Bb-CYCT, Bj-CYCS, and Bj-CYCT with heme were predicted by AlphaFold3 (Abramson et al. 2024). The resulting structures were visualized using PyMOL (Schrödinger LLC, USA).

### Gene cloning

Each *cyc* gene encoding Bb-CYCS, Bb-CYCT, Bj-CYCS, and Bj-CYCT was synthesized (ThermoFisher Scientific, USA) with codon optimization for *E. coli*, which had been fused with the signal peptide gene of *Pseudomonas aeruginosa* cytochrome *c*_551_ at the N-terminus for periplasmic protein localization (Sambongi and Ferguson 1994). The synthesized genes were cloned into the multicloning site between EcoRI and SalI restriction sites of a modified pKK223-3ΔSP vector (with a deletion between SphI and PvuII restriction sites), downstream of the *tac* promoter, using the In-Fusion cloning system (Takara Bio, Japan).

A plasmid for expression of the Bj-CYCT Y46F variant was also prepared with the PrimeStar Max Remix kit (Takara Bio, Japan), which was applied to the Bj-CYCT wild-type gene inserted into the pKK223-3ΔSP vector as mentioned above. Tyr-46 in Bj-CYCT was substituted with Phe, which is the corresponding residue in Bb-CYCS and Bj-CYCS. The primer sequences were as follows: Fw: 5′-GCAGGTTTTAGTTATACCGATGCGAAT-3′ and Rv: 5′-ATAACTAAAACCTGCTGCCTGACCTGT-3′. The correct introduction of the mutation was confirmed by DNA sequencing.

### Protein expression and purification

The plasmids carrying genes for each of 4 CYC wild-types and the Bj-CYCT Y46F variant were introduced into *E. coli* strain JCB387 containing the pEC86 plasmid carrying the genes for CYC maturation (*ccmA-H* genes). The *E. coli* strain JCB387 cells expressing CYC were initially pre-cultured in liquid LB medium containing 100 µg/mL ampicillin and 30 µg/mL chloramphenicol. These LB pre-cultures (2 mL) were inoculated into 20 mL LB medium containing the same antibiotics, followed by shaking at 37 °C for 4 h. After this pre-cultivation, 20 mL LB medium was inoculated into 2 L minimal medium (Sambongi and Ferguson 1994) containing the same antibiotics, 20% (v/v) glycerol, and 1 mg/mL 5′-aminolevulinic acid in a 5 L flask, and then the cells were aerobically grown at 37 °C with shaking for 21 h.

The following purification steps were performed at 4 °C. Periplasmic protein fractions containing the recombinant CYC proteins were extracted by the cold osmotic shock method with the addition of 20% (w/v) sucrose solution and then followed by the addition of distilled water (Neu and Heppel 1965; Oikawa et al. 2005). The periplasmic protein fractions containing CYC proteins were dialyzed against 10 mM phosphate buffer (pH 7.0) and concentrated using an Amicon Ultra-15 centrifuge filter (Merck, Germany). The concentrated protein solutions were loaded onto a HiTrap SP cation-exchange column (HiTrap SP, 1.6 cm in diameter × 2.5 cm in height, Cytiva, USA) that had been equilibrated with the same buffer. The protein fractions were eluted using a linear NaCl gradient (0 to 150 mM). The resulting fractions were dialyzed against 10 mM phosphate buffer (pH 7.0) with 150 mM NaCl and loaded onto a Superdex75 gel filtration column (1.6 cm in diameter × 60 cm in height, GE Healthcare, USA) that had been equilibrated with the same buffer. The final protein samples were dialyzed against Milli-Q water and concentrated. The protein purity was checked by SDS-PAGE with Coomassie brilliant blue staining. The CYC concentration was determined by the extinction coefficient at 551 nm of pyridine ferrohemochrome (Berry and Trumpower 1987). The N-terminal sequences of the purified CYC proteins were determined via Edman degradation to verify correct cleavage of the periplasmic targeting signal peptide.

### Crystallization, X-ray diffraction, and structure determination

The crystallization conditions for the Bj-CYCS protein were screened using the Wizard Classic crystal screening kit 1-4 (Rigaku, Japan) on the 96-well in situ MiTeGen plate (MiTeGen, USA) at 20 °C in a sitting drop vapor diffusion method. The protein was dissolved in Milli-Q water at a concentration of 20 mg/mL. The reservoir solution conditions for the crystallization are as follows: 40% polyethylene glycol (PEG) 400, 0.1 M Tris-HCl buffer (pH 8.5), 0.2 M lithium sulfate, comprising 100 nL protein solution and 100 nL reservoir solution. The protein crystals were observed after 2 d.

Initial diffraction analysis at room temperature of crystals to establish the space group and unit cell was conducted at the VMXi beamline of the Diamond Light Source (Didcot, UK), resulting in a P41 21 2 space group with diffraction extending to 3.2 Å resolution. Subsequently, X-ray diffraction data were collected from Bj-CYCS crystals at 100 K, with 10% glycerol as a cryoprotectant, at the I03 beamline of the Diamond Light Source using a wavelength of 0.976 Å. Data processing was carried out using Xia2 and DIALS (Winter 2010). The crystal structure was solved by molecular replacement through the Dimple pipeline (Wojdyr et al. 2014) using a monomer from an AlphaFold2 model as the search template. Structural refinement was conducted using REFMAC5 (Murshudov et al. 2011), and manual model building was performed with COOT (Emsley and Cowtan 2004) within the CCP4 software suite. The structure was validated using MolProbity (Chen et al. 2010). Figures were generated using PyMOL (Schrödinger LLC, USA). The parameters for X-ray diffraction were summarized in Table 1. The crystal structure has been deposited in the Protein Data Bank under PDB ID 24OQ.

**Table 1:** X-ray data collection, structure solution, and refinement of Bj-CYCS.

	Bj-CYCS
Beamline	I03
Resolution range (Å)	51.23 to 2.48 (2.52 to 2.48)
Space group	P 41 21 2
Unit cell (Å, °)	72.43 72.43 35.91 90.00 90.00 90.00
Unique reflections	3,436 (42)
Multiplicity	30.5 (1.2)
Completeness (%)	93.0 (23.6)
Mean I/sigma(I)	37.1 (2.7)
R-merge	0.078 (0.248)
R-pim	0.013 (0.230)
CC(1/2)	1.0 (0.6)
R-work	0.210
R-free	0.289
Protein residues	101 (3 amino acids at the C-terminus are omitted)
RMSD (bonds, Å)	0.010
RMSD (angles, °)	2.475
Ramachandran favored (%)	95.9
Average *B*-factor (Å^2^)	41.5
Coordinate file	AlphaFold2 model
PDB ID	24OQ

### Spectroscopic analysis

UV-visible absorption spectra of the recombinant CYC proteins were measured in the air-oxidized and reduced states. Approximately 9 µM recombinant CYC protein was dissolved in 10 mM potassium phosphate buffer (pH 7.0) and analyzed at 25 °C using a JASCO V-730 Bio spectrophotometer (JASCO, Japan). The oxidized forms were prepared by exposure to air, and reduced forms were obtained by adding a grain of sodium dithionite. The spectra were recorded in the range from 350 to 600 nm.

To evaluate H_2_O_2_ resistance, ∼5 µM oxidized CYC proteins were mixed with 3 mM H_2_O_2_ in 10 mM potassium phosphate buffer (pH 7.0). The absorbance at 408 nm was monitored continuously for 60 min at 25 °C. Heme degradation rates were calculated as the percentage decrease in absorbance from time 0 (Liu et al. 2006). Means with standard deviations of the rates were obtained from 3 independent measurements.

CD spectra were recorded using a JASCO J-820 CD spectrometer (JASCO, Japan). The CYC proteins (20 µM) were dialyzed in 10 mM potassium phosphate buffer (pH 7.0) and analyzed in a 1 mm pathlength cuvette. The CD signals in the range from 190 to 260 nm were collected at 25 °C.

### Thermal denaturation measurement

Thermal denaturation of the recombinant CYC proteins was measured by monitoring temperature-induced changes in CD ellipticity at 222 nm, which is a characteristic wavelength for α-helical structure. Measurements were performed using a JASCO J-820 CD spectrometer (JASCO, Japan) equipped with a pressure-resistant cell holder. The CYC protein samples (20 µM) were dissolved in 10 mM potassium phosphate buffer (pH 7.0) and placed in a 1 mm pathlength cuvette. The temperature was increased from 25 to 140 °C at a rate of 1.0 °C/min under a constant pressure of 0.9 MPa to prevent boiling. The CD ellipticity values at 222 nm were recorded at 0.5 °C intervals.

Raw data were corrected for baseline drift, and the data from 30 to 140 °C were normalized to calculate the fraction of denatured protein as described previously (Uchiyama et al. 2002). These values were plotted against temperature, and the resulting thermal denaturation curves were fitted using nonlinear least-squares regression to determine the denaturation temperature (*T*_m_), defined as the midpoint of the unfolding transition. Means with standard deviations of the *T*_m_ values were obtained from 5 independent measurements.

### Cyclic voltammetry

Redox potential values of the CYC proteins were determined by cyclic voltammetry using a Potentiostat-Galvanostat PGSTAT12 (ECO CHEMIE, Utrecht, The Netherlands) as described previously (Takeda et al. 2009). A glassy carbon electrode (GCE) was used as the working electrode and polished with a 0.05 µm alumina slurry prior to each use. Approximately 2 μL of protein solutions (∼1 mM) dissolved in 20 mM HEPES–KOH buffer (pH 7.0) were spread onto the GCE surface and covered with a semi-permeable membrane.

A saturated calomel electrode (SCE) served as the reference electrode, and a platinum wire was used as a counter electrode. All electrodes were set in 20 mM HEPES–KOH buffer (pH 7.0) saturated with N_2_ gas, and the cyclic voltammogram was measured at 25 °C. The potential was swept from −300 to +100 mV vs. SCE at a scan rate of 10 mV s^−1^. Voltammograms were recorded, and the midpoint redox potential (*E*_m_) value was calculated as the average of the anodic and cathodic peak potentials. All potentials were converted to the standard hydrogen electrode (SHE) scale using the formula: *E*_SHE_ = *E*_SCE_ + 241.2 mV (at pH 7.0 and 25 °C). Redox measurements were independently carried out 3 times, and the *E*_m_ values are presented as means with standard deviations.

## Supplementary Material

msag243_Supplementary_Data

## Data Availability

The transcriptome sequencing data generated in this study have been deposited in the DDBJ Sequence Read Archive under accession numbers DRR911195 and DRR911196. The crystal structure of Bj-CYCS has been deposited in the Protein Data Bank under accession code 24OQ. All other data supporting the findings of this study are available within the article and its Supplementary material.
