# Supplementary material for: Ancient nuclear genome duplication events in vertebrates facilitated the functional diversification of ohnologous cytochrome c: insights from Buergeria frogs
Source: Mol Biol Evol. 2026 Sep 25;43(10):msag243. doi: 10.1093/molbev/msag243 (PMC13655612; doi:10.1093/molbev/msag243)
Supplement: Supplementary file 1 [file msag243_supplementary_data.docx]

**Supplementary data**

**Ancient Nuclear Genome Duplication Events in Vertebrates Facilitated the Evolution of Hydrogen Peroxide Resistance of Cytochrome *c*: Insights from *Buergeria* Frogs**

Yuka Asaeda,^1,2^ Sotaro Fujii,^1^* Hajime Ogino,^1,2^ Michael A. Hough,^3^ Takeshi Igawa,^1,2^* Yoshihiro Sambongi^4^

^1^ Graduate School of Integrated Sciences for Life, Hiroshima University, Higashi-Hiroshima, Japan

^2^ Amphibian Research Center, Hiroshima University, Higashi-Hiroshima, Japan

^3^ Diamond Light Source, Harwell Science and Innovation Campus, Didcot, UK

^4^ Faculty of Human Environmental Studies, Hiroshima Shudo University, Hiroshima, Japan

**2.5 Å**

**Fig. S1**

Expression levels of *X. tropicalis cycs* and *cyct* genes in somatic tissues and ovary from female (N=3) and in testis from male (N=3) based on transcriptome data from NCBI Sequence Read Archive with accession number DRR540384- DRR540573.

The tissues used are adipose cell (AD), blood (BL), bone marrow (BM), brain (BR), eye (EY), heart (HE), small intestine (IN), kidney (KI), liver (LI), lung (LU), muscle (MU), ovary (OV), pancreas (PA), skin (SK), spleen (SP), and testis (TE). Raw RNA-seq reads were subjected to quality control and adapter trimming using Trimmomatic v0.39 (10.1093/bioinformatics/btu170). Low-quality bases and adapter sequences were removed using the following parameters: ILLUMINACLIP (adapter sequences): 2:30:10, LEADING:3, TRAILING:3, SLIDINGWINDOW:4:15, and MINLEN:36. This ensured that only high-quality reads were retained for downstream analysis. The quality-filtered reads were aligned to the *X. tropicalis* reference genome (Xt10/xenTro10) using STAR aligner v2.7.10a (10.1093/bioinformatics/bts635). STAR was run in two-pass mode to improve alignment accuracy, particularly for novel splice junctions. The genome index was generated using the reference genome FASTA file and gene annotation GTF file with default parameters. Alignment was performed with the default parameters. Gene expression levels were quantified using RSEM v1.3.3 (10.1186/1471-2105-12-323). RSEM was used to estimate transcript and gene abundances from the STAR-aligned BAM files. The RSEM reference was prepared using the *X. tropicalis* Xt10 genome and corresponding gene annotation. The expression level was calculated and normalized as transcripts per million (TPM) value. The asterisks indicate the statistically significant differences as determined by t-test (*: *p*<0.05, **: *p*<0.01).

**Fig. S2**

SDS-PAGE for the four recombinant *Buergeria* CYC proteins after purification.

**Fig. S3**

Spectroscopic and electrochemical characterization of the recombinant Bj-CYCT Y46F variant. (A) Visible absorption spectra of the protein at 25 °C. Orange and blue lines represent the spectra of oxidized and reduced forms, respectively. Relevant wavelengths are indicated. (B) CD spectra of the protein at 25 °C. Relevant wavelengths are indicated. (C) Normalized thermal denaturation curve by CD analysis based on CD ellipticity at 222 nm. The fractions of representative raw data are shown in circles at temperature intervals of 2.5 °C. The fitting curves are also shown in solid black lines. Measurement was performed five times. (D) Cyclic voltammogram by CV analysis. The representative cyclic voltammogram for each protein is shown. Measurement was performed three times.

**Table S1**

Structural comparison between Bj-CYCS and other CYC proteins.

| Organism | PDB ID | Identity | RMSD* (Å) |
| --- | --- | --- | --- |
| *Rattus norvegicus* | 5C0Z | 94/104 (90%) | 0.410 |
| *Bos taurus* | 2B4Z | 92/103 (89%) | 0.457 |
| *Equus caballus* | 1HRC | 91/104 (86%) | 0.373 |
| *Mus musculus* | 2AIU | 89/104 (86%) | 0.366 |
| *Homo sapiens* | 3ZCF | 88/104 (84%) | 0.372 |
| *Thunnus thynnus* | 1I54 | 88/103 (85%) | 0.362 |
| *Saccharomyces cerevisiae* | 1YFC | 62/102 (61%) | 0.974 |
| *Paracoccus denitrificans* | 1QL3 | 42/98 (43%) | 0.719 |

*RMSD: root-mean-square deviation, based on Cα atoms of the main chain.

**Table S2**

PCR primers used for qRT-PCR.

| Primer | Sequence (5’ to 3’) |
| --- | --- |
| *Bb-CYCS*_F | AAAGTACATCCCCGGCACAAAGATGATC |
| *Bb-CYCS*_R | CAGGAAGTTGAGCTTTTGAGATACGC |
| *Bb-CYCT*_F | CAAAGGTATTGTGTGGGGAGAGGACACTC |
| *Bb-CYCT*_R | AATAGGCTATTAGATCTACGCGCTCG |
| *tuba4a*_F | GCTTATCATGAGCAGCTTTCTGTGTCTGAG |
| *tuba4a*_R | GTATTTACCGTGTCTGGGGTCACATTTCAC |

*F and R indicate forward and reverse, respectively.

**Table S3**

Quality of RNA samples from *B. buergeri*.

| Female | Individual | Conc. (ng/ul) | A260 | A280 | 260/280 | 260/230 |
| --- | --- | --- | --- | --- | --- | --- |
| Ovary | 1 | 274.1 | 6.853 | 3.498 | 1.96 | 0.52 |
|  | 2 | 144.7 | 3.617 | 2.171 | 1.67 | 0.25 |
|  | 3 | 152.1 | 3.804 | 2.305 | 1.65 | 0.28 |
| Muscle | 1 | 565.5 | 14.139 | 7.389 | 1.91 | 0.39 |
|  | 2 | 379.7 | 9.493 | 4.971 | 1.91 | 0.59 |
|  | 3 | 385.1 | 9.627 | 5.015 | 1.92 | 0.60 |
| Heart | 1 | 298.6 | 7.465 | 4.014 | 1.86 | 0.65 |
|  | 2 | 476.2 | 11.906 | 6.198 | 1.92 | 0.76 |
|  | 3 | 457.3 | 11.432 | 5.910 | 1.93 | 0.79 |
|  |  |  |  |  |  |  |
| Male | Individual | Conc. (ng/ul) | A260 | A280 | 260/280 | 260/230 |
| Testis | 1 | 1010.1 | 25.252 | 12.891 | 1.82 | 0.42 |
|  | 2 | 873.2 | 21.829 | 12.413 | 1.76 | 0.39 |
|  | 3 | 219.9 | 5.498 | 3.058 | 1.80 | 0.44 |
| Muscle | 1 | 1586.7 | 39.668 | 20.343 | 1.95 | 0.85 |
|  | 2 | 415.9 | 10.397 | 5.319 | 1.95 | 0.73 |
|  | 3 | 510.7 | 12.768 | 8.104 | 1.58 | 0.31 |
| Heart | 1 | 420.2 | 10.505 | 5.423 | 1.94 | 0.69 |
|  | 2 | 503.5 | 12.589 | 7.481 | 1.68 | 0.35 |
|  | 3 | 643.6 | 16.090 | 9.332 | 1.72 | 0.35 |

**Table S4**The *C*_t_ value in qRT-PCR (n=3).

| Female | Individual | *Bb-CYCS* | *Bb-CYCT* | *tuba4a* |
| --- | --- | --- | --- | --- |
| Ovary | 1 | 19.0 | 25.3 | 24.2 |
|  | 2 | 22.0 | 27.9 | 24.7 |
|  | 3 | 22.7 | 27.5 | 24.6 |
| Muscle | 1 | 20.6 | 22.7 | 23.9 |
|  | 2 | 21.0 | 25.4 | 24.2 |
|  | 3 | 19.9 | 25.3 | 24.6 |
| Heart | 1 | 20.9 | 25.6 | 24.5 |
|  | 2 | 20.3 | 26.2 | 24.5 |
|  | 3 | 21.0 | 27.2 | 24.6 |
|  |  |  |  |  |
| Male | Individual | *Bb-CYCS* | *Bb-CYCT* | *tuba4a* |
| Testis | 1 | 25.8 | 25.3 | 24.8 |
|  | 2 | 23.2 | 23.2 | 24.4 |
|  | 3 | 23.3 | 22.7 | 24.2 |
| Muscle | 1 | 23.6 | 27.2 | 24.6 |
|  | 2 | 21.4 | 24.7 | 24.2 |
|  | 3 | 22.4 | 25.5 | 24.4 |
| Heart | 1 | 18.8 | 23.3 | 24.7 |
|  | 2 | 21.8 | 25.6 | 24.8 |
|  | 3 | 22.3 | 26.7 | 24.8 |
